# Zeb1 Is a Determinant of EMT in Human MDA-MB-231 Breast Cancer Cells and M13-MDA231 Tumor Hybrids

**DOI:** 10.3390/ijms27188248

**Published:** 2026-09-16

**Authors:** Ida Friederike Wörner, Thomas Dittmar

**Affiliations:** Immunology and Tumor Biology, Center for Biomedical Education and Research (ZBAF), Witten/Herdecke University, 58453 Witten, Germany; ida.woerner@uni-wh.de

**Keywords:** breast cancer, EMT, cell fusion, Zeb1

## Abstract

Zeb1 is a well-known epithelial–mesenchymal transition (EMT) transcription factor and is highly expressed in aggressive cancers. Tumor hybrids, which were derived from fusion events between cancer cells and normal cells, such as macrophage and stem cells, can possess novel properties, such as enhanced metastatic activity. The role of Zeb1 was studied in M13-MDA231-6 and -13 tumor hybrids that were derived from spontaneous fusion events between MDA-MB-231-Hyg human breast cancer cells and M13SV1-EGFP-Neo human breast epithelial cells. The stable CRISPR/Cas9-mediated Zeb1-KO was correlated with re-expression of E-Cadherin in MDA-MB-231-Hyg-Zeb1-KO and M13-MDA231-13-Zeb1-KO cells, but not in M13-MDA231-6-Zeb1-KO cells. Similarly, the proliferation of MDA-MB-231-Hyg-Zeb1-KO in M13-MDA231-13-Zeb1-KO cells, but not in M13-MDA231-6-Zeb1-KO cells, was decreased as compared to non-edited cells. The migratory activity of Zeb1-KO cells was markedly reduced in transmigration and invasion studies compared to non-edited cells. However, MDA-MB-231-Hyg-Zeb1-KO cells and M13-MDA231-6-Zeb1-KO tumors only showed reduced migratory behavior in a scratch/wound healing assay, while M13-MDA231-13-Zeb1-KO exhibited enhanced locomotory activity. In contrast, no clear effects of Zeb1 knockout were observed on colony-forming capacity or CD44+/CD104+ expression. In summary, our data support the role of Zeb1 as a driver of EMT and cancer cell migration.

## 1. Introduction

Epithelial-to-mesenchymal transition (EMT) is considered the initial step in cancer metastasis [1,2,3,4]. It represents a highly complex and well-coordinated process in which cancer cells lose stepwise epithelial features; for example, E-Cadherin facilitated cell–cell contact while concomitantly acquiring a more mesenchymal-like phenotype [1,2,3,4]. Therefore, cancer cells that have passed through the EMT process can disseminate from the primary tumor to start their long march until they have finally reached distant organs and initiate metastases formation [1,2,3,4].

The process of EMT is controlled by so-called core EMT regulatory networks, which consists of the interplay of EMT transcription factors, microRNAs, post-translational modifications, and alternative splicing [3,4]. A variety of EMT-inducing triggers have been identified, such as transforming growth factor-β (TGF-β), Wnt proteins, cytokines, ECM–integrin interactions, and hypoxia [3,4]. Regarding the core EMT regulatory networks, the interplay of two microRNAs (miR-34a-5p and miR-200c-3p) and two EMT transcription factors (Snail and Zeb1) became of interest. They form two interconnected mutually inhibitory feedback loops (miR-34a-5p-Snail and miR-200c-3p-Zeb1) that direct whether cancer cells remain in an epithelial (E), mesenchymal (M), or mixed epithelial/mesenchymal (E/M) state [5,6]. Notably, this mixed E/M state, which has also been named the hybrid E/M or quasi-mesenchymal state [3,7], does not define an intermediate state that cancer cells have to acquire during EMT but is a stable phenotype that cancer cells can adopt [5,6,7,8,9,10,11]. Additionally, it was demonstrated that cancer cells with a mixed E/M phenotype exhibited potential cancer stem cell (CSC) properties [10,12,13]. Jolly and colleagues demonstrated that the mixed E/M state is chiefly regulated via the miRNA-200c-3p-Zeb1 feedback loop [5,6,11]. These results indicate that the EMT transcription factor Zeb1 may also be a determinant of stemness characteristics in cancer cells.

In any case, Zeb1 is commonly known as a driver of EMT due to its role in down-regulating the classical epithelial marker E-Cadherin [1,2,3,4]. Elevated levels of Zeb1 expression have been identified in a variety of human cancers, including colon cancer [14], glioblastoma [15], and breast cancer [16,17,18]. Moreover, elevated Zeb1 expression levels were further associated with disease prognosis, increased metastatic spreading, and an overall poorer prognosis [14,15,16,17,18,19]. For example, TGF-β-induced mesenchymal differentiation of glioblastoma cell lines was associated with morphological changes, enhanced mesenchymal marker expression, migration, and invasion in vitro [15]. Moreover, TGF-β-responding glioblastoma neurospheres formed invasive tumors in mice and revealed mesenchymal marker expression in immunohistochemical analyses [15]. Graham and colleagues showed that Zeb1 and androgen receptor (AR) crosstalk in triple-negative breast cancer (TNBC) [17]. Zeb1 directly induced the expression of AR in TNBC cell lines [17]. Similarly, blockade of AR signaling with bicalutamide resulted in suppression of ZEB1 protein expression in two TNBC cell lines [17]. Moreover, the shRNA-mediated knock-down of AR inhibition was correlated with markedly deceased migratory activity towards dihydrotestosterone [17]. Thus, cell migration in TNBC cells might be related to Zeb1 and AR crosstalk. Similarly, Mohammadi Ghahhari et al. demonstrated that the functional interaction between estrogen receptor-α and Zeb1 may alter the tissue tropism of metastatic breast cancer cells towards bone.

The biological phenomenon of cell fusion describes the merger of the plasma membranes of two (and more) cells that result in the formation of bi- and multi-nucleated hybrid cells [20,21,22,23]. Cell fusion is essential for physiological processes, such as fertilization, placentation, and myogenesis [20,21,22,23]. However, the process also plays a crucial role in pathophysiological processes, including the infection of host cells with enveloped viruses and the merger of cancer cells [20,21,22,23]. The merger of cancer cells and normal cells, such as macrophage and stem cells, results in the formation of tumor hybrids, which often possess novel properties, including increased metastatic behavior [20,21,22,23,24,25,26]. In a previous work, we demonstrated that Zeb1-KO in M13HS tumor hybrids, which were derived from M13SV1-EGFP-Neo human breast epithelial cells and HS578T-Hyg human breast cancer cells through spontaneous fusion [27], was correlated with differential migratory phenotypes. Although all Zeb1-KO cells exhibited reduced transmigration rates compared to untreated cells, the invasive capabilities of both cell types remained largely similar [27]. Interestingly, Zeb1-KO was not correlated with re-induction of E-Cadherin expression in HS578T-Hyg breast cancer cells and M13HS tumor hybrids. It is unknown whether this was due to breast cancer cell line- and tumor hybrid-specific characteristics.

In the present study, we investigated the impact of Zeb1-KO on M13-MDA231 tumor hybrids, which were derived from M13SV1-EGFP-Neo human breast epithelial cells and MDA-MB-231-Hyg human breast cancer cells through spontaneous fusion [28]. Indeed, Zeb1-KO was correlated with re-induction of E-Cadherin expression in MDA-MB-231-Hyg breast cancer cells and one M13-MDA231 hybrid clone. Moreover, all Zeb1-KO cells exhibited decreased transmigration and invasion capacity, supporting the role of Zeb1 in EMT and cancer cell migration.

## 2. Results

### 2.1. Successful CRISPR/Cas9-Mediated Zeb1-KO in MDA-MB-231-Hyg Breast Cancer Cells and M13-MDA231 Hybrids

**Figure 1:**
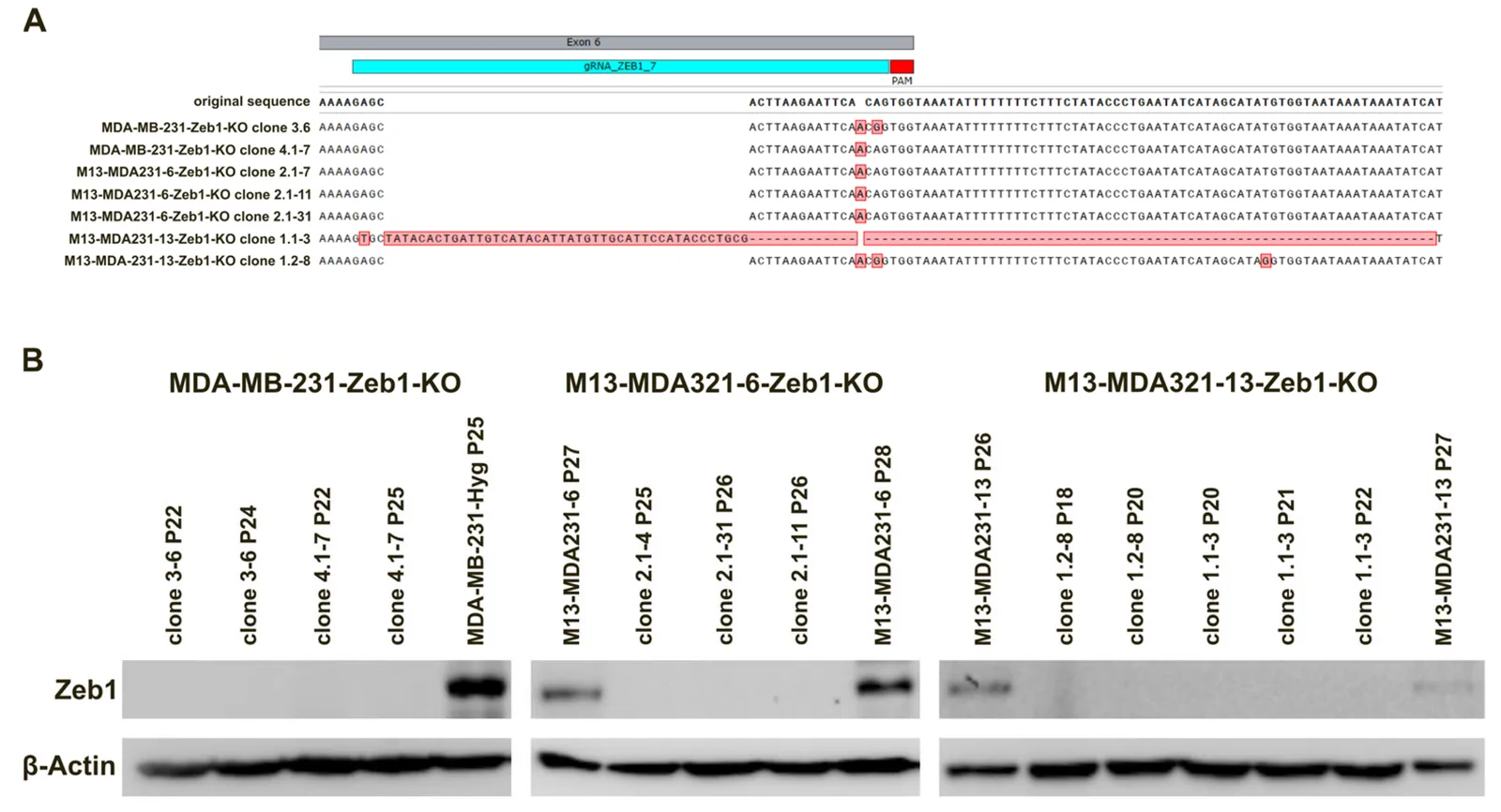
Successful CRISPR/Cas9-mediated Zeb1-KO in MDA-MB-231-Hyg breast cancer cells and M13-MDA231 hybrid. (**A**) Alignment of the obtained Sanger sequencing results of Zeb1-KO cells with the original Zeb1 sequence. The Zeb1-specific gRNA sequence targets a region in exon 6 of the Zeb1 gene. The areas marked in red indicate the edited regions of the genomes of individual Zeb1-KO clones. (**B**) Representative Western blot results of different passages of various Zeb1-KO clones. Non-edited cells were used as a positive control to demonstrate successful Zeb1-KO.

Zeb1-KO in MDA-MB-231-Hyg breast cancer cells and M13-MDA231 hybrids was performed in accordance with the work of Merckens and colleagues [29]. The results are summarized in Figure 1 and clearly show stable Zeb1-KO in different passages and clones of the edited genome. M13SV1-EGFP-Neo cells lack Zeb1 expression [29]. Therefore, no CRISPR/Cas9 genome editing was performed. Stable Zeb1-KO clones were pooled for further analyses.

### 2.2. E-Cadherin Is Re-Expressed in MDA-MB-231-Hyg-Zeb1-KO Cells and M13-MDA231-13-Zeb1-KO Hybrid Cells

**Figure 2:**
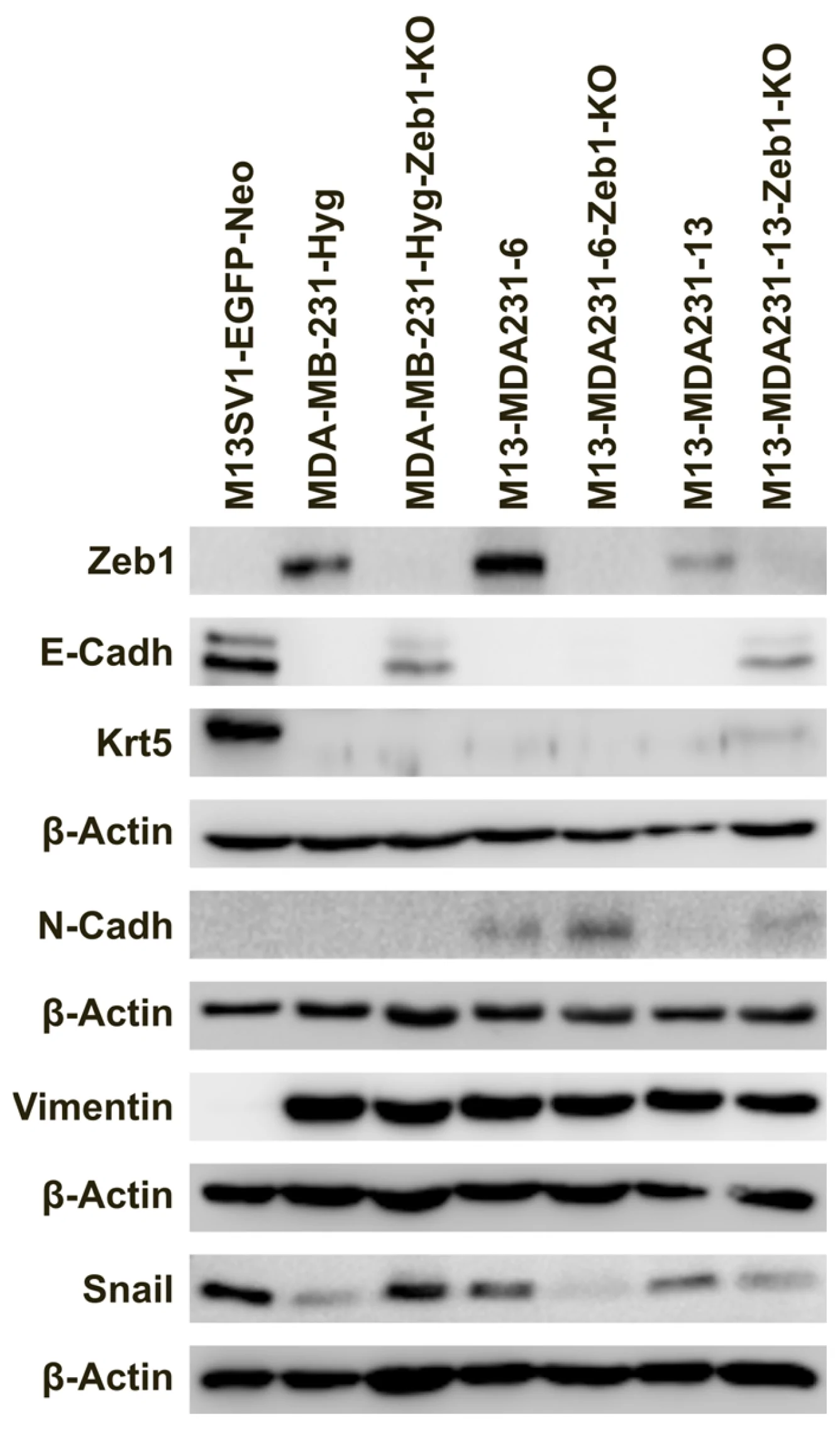
E-Cadherin is re-expressed in MDA-MB-231-Hyg-Zeb1-KO cells and M13-MDA231-13-Zeb1-KO hybrid cells. Representative Western blot data of at least three independent experiments are shown. Please note that some blots, such as Zeb1, E-Cadherin, and Cytokeratin 5 (Krt5), were triple stained and thus have identical β-actin bands.

Zeb1 is a well-known EMT transcription factor that represses the expression of epithelial-specific genes, such as E-Cadherin [30]. Therefore, Zeb1 up-regulation is consistent with the loss of E-Cadherin represents an initial step in the EMT process [30,31]. We have already demonstrated that the epithelial and mesenchymal gene expression pattern of stable Zeb1-KO in HS578T-Hyg breast cancer cells and M13HS hybrids remained unchanged [29]. In fact, we only observed up-regulation of the EMT transcription factor Snail in Zeb1-KO cells, while the expression of classical epithelial markers, such as E-Cadherin, and mesenchymal markers, like N-Cadherin and Vimentin, was not altered [29]. Therefore, the expression of epithelial markers (E-Cadherin and Cytokeratin 5 (Krt5)), mesenchymal markers (N-Cadherin and Vimentin), and Snail was analyzed by Western blot in non-edited cells and Zeb1-KO variants (Figure 2).

Interestingly, the overall effect of Zeb1-KO on the epithelial and mesenchymal gene expression pattern was rather moderate. Notably, E-Cadherin was re-expressed in MDA-MB-231-Hyg-Zeb1-KO cells and M13-MDA231-13-Zeb1-KO hybrid cells, but not in M13-MDA231-6-Zeb1-KO hybrids (Figure 2). Interestingly, Zeb1-KO was associated with slightly higher N-Cadherin expression in both Zeb1-KO hybrid clones despite having markedly lower Snail expression levels (Figure 2). This finding was rather unexpected since Snail is another well-known EMT transcription factor that favors the induction of EMT [32]. Therefore, lower Snail expression levels in M13-MDA231-Zeb1-KO hybrids should be rather associated with lower N-Cadherin expression levels. In contrast, Zeb1-KO is associated with higher Snail expression levels in MDA-MB-231-Hyg cells (Figure 2), which is consistent with our previous data [29]. The results for Cytokeratin 5 (Krt5) are not quite clear. Western blot results likely indicate a very faint Cytokeratin 5 (Krt5) expression in M13-MDA231-13-Zeb1-KO hybrid cells, which was not observed in MDA-MB-231-Hyg-Zeb1-KO and M13-MDA231-6-Zeb1-KO hybrid cells (Figure 2). Vimentin expression levels remained unchanged in Zeb1-KO cells (Figure 2), which is also consistent with previous data [29].

### 2.3. Zeb1-KO Has a Differential Effect on Cells’ Proliferation Rate

**Figure 3:**
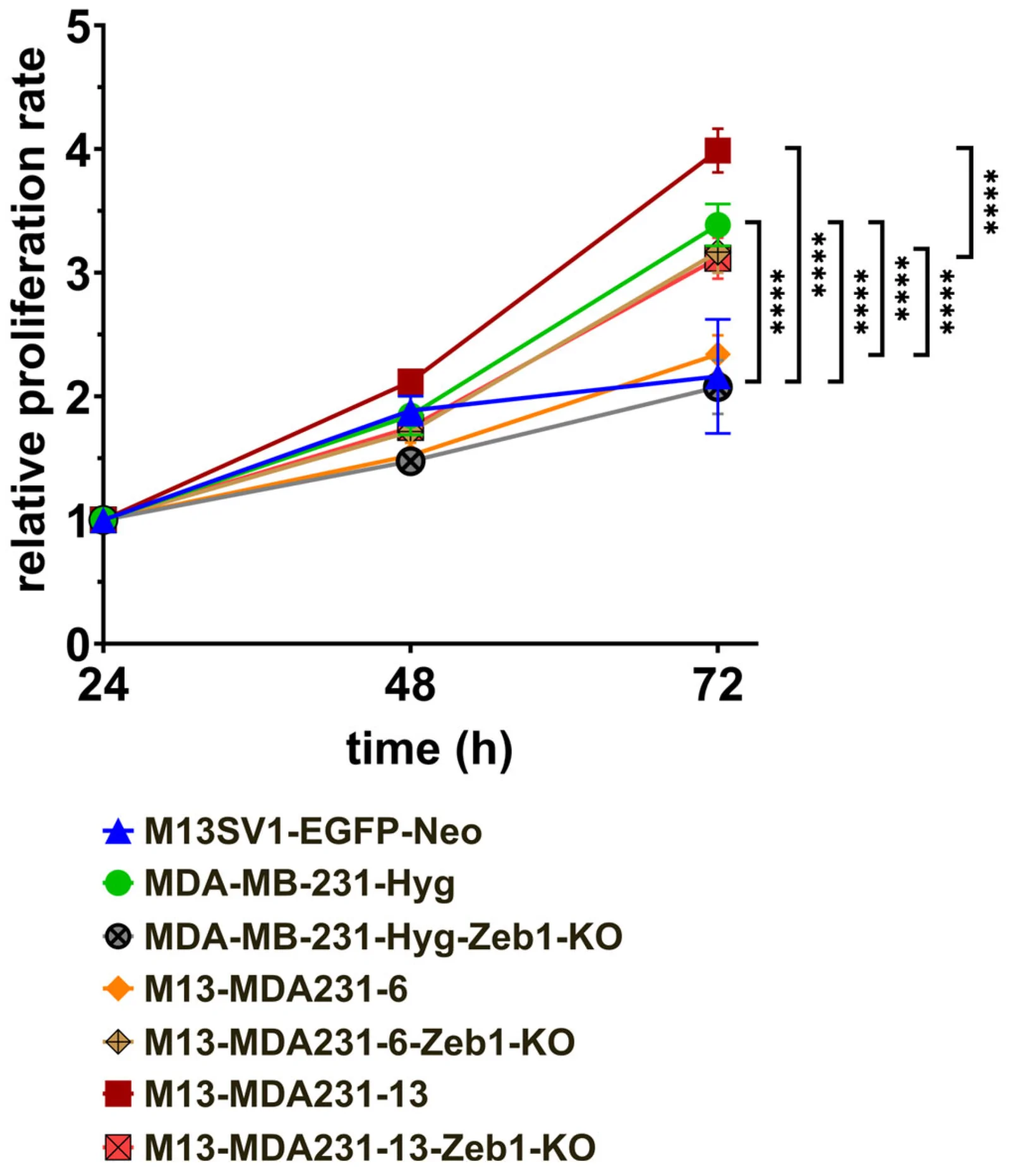
Zeb1-KO has a differential effect on the cells’ proliferation rate. The mean ± S.E.M. of at least three independent experiments are shown. The relative proliferation rate of the cells, which was set to 1, was calculated in relation to 24 h. Statistical significance was calculated using a Two-Way ANOVA and Tukey’s post hoc test. **** = *p* < 0.0001.

Zeb1 is not only important for the EMT process and, thus, for a more motile and invasive phenotype, but is also associated with cell proliferation [31,33]. Therefore, we used a CCK8 proliferation assay and found that Zeb1-KO has a differential effect on the cells’ proliferation rate, as shown in Figure 3.

The proliferation rates of MDA-MB-231-Hyg-Zeb1-KO cells and M13-MDA231-13-Zeb1-KO hybrids were significantly lower as compared to their parental counterparts (Figure 3). For example, the relative proliferation rate of MDA-MB-231-Hyg breast cancer cells after 72 h was about 3.39 ± 0.17, while that of MDA-MB-231-Hyg-Zeb1-KO cells significantly decreased to 2.08 ± 0.22 (*p* < 0.0001; Figure 3). Similarly, the relative proliferation rate of M13-MDA231-13-Zeb1-KO hybrids was significantly lower (3.12 ± 0.17; *p* < 0.0001) compared to that of parental M13-MDA231-13 hybrids (3.99 ± 0.18; Figure 3). On the other hand, the relative proliferation rate of M13-MDA231-6-Zeb1-KO hybrids was significantly higher (3.17 ± 0.17; *p* < 0.0001) compared to that of parental M13-MDA231-6 hybrids (2.34 ± 0.15; Figure 3).

### 2.4. Zeb1-KO Has a Differential Effect on the Cells’ Colony Formation Capacity

**Figure 4:**
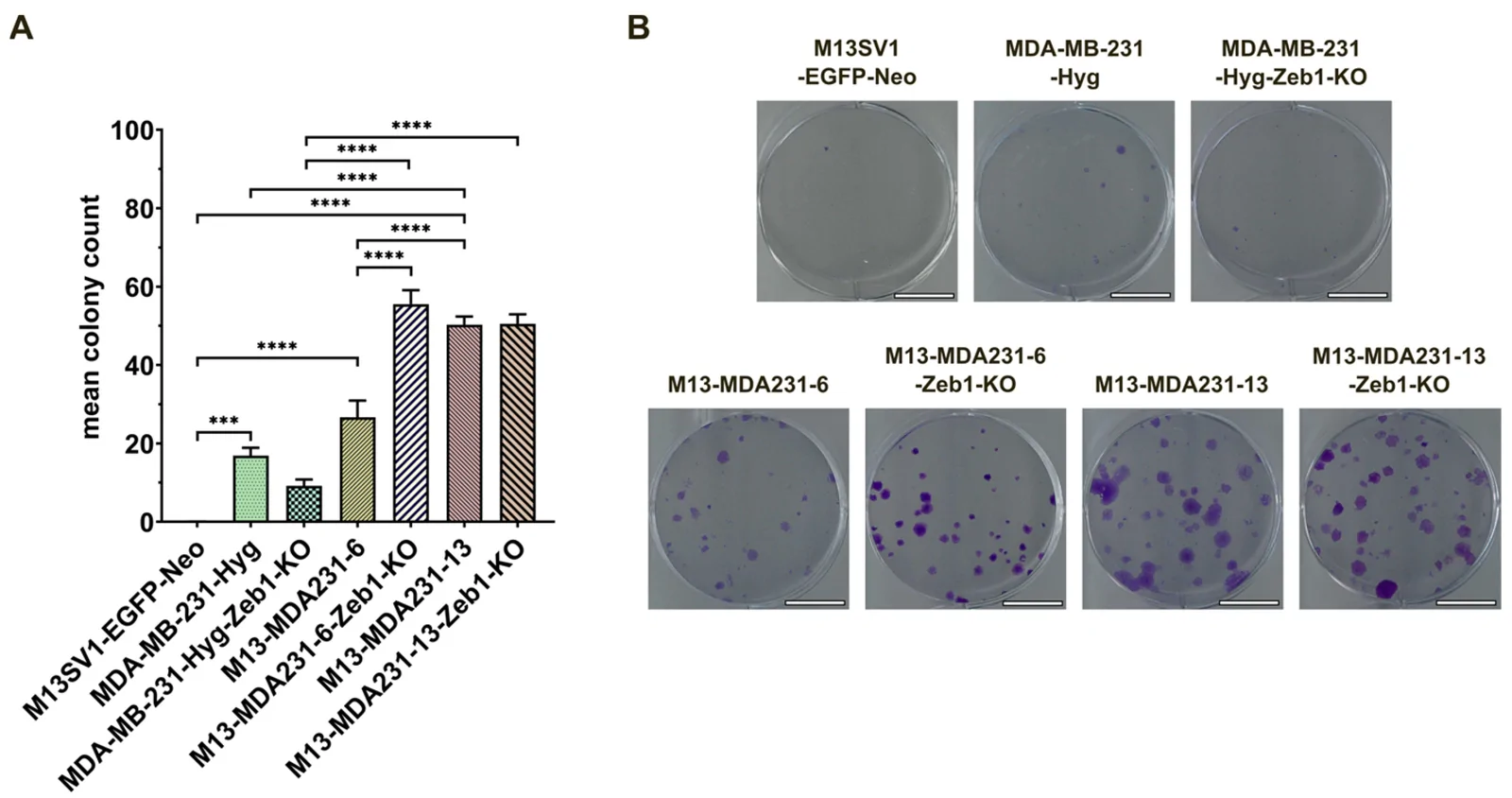
Zeb1-KO has a differential effect on the cells’ colony formation capacity. (**A**) The mean ± S.E.M. of at least three independent experiments. Statistical significance was calculated using an ordinary one-way ANOVA and Tukey’s post hoc test. *** = *p* < 0.001; **** = *p* < 0.0001. (**B**) Representative images of colony formation assays. Bar = 100 µm.

Next, we analyzed the impact of Zeb1-KO on the cells’ capacity to from colonies. In accordance with previous data [29,34], M13SV1-EGFP-Neo cells had a rather low colony formation capacity. In fact, only one colony was formed in three independent experiments. In contrast, the colony formation capacity of MDA-MB-231-Hyg cells and M13-MDA231 hybrids was significantly higher (Figure 4A). M13-MDA231 hybrids exhibited a higher colony formation capacity (M13-MDA231-6: 27 ± 4 colonies; M13-MDA231-13: 50 ± 2 colonies; Figure 4A) compared to MDA-MB-231-Hyg cells (MDA-MB-231-Hyg: 17 ± 2 colonies; Figure 4A).

Interestingly, the Zeb1-KO yielded in different results. For example, the colony formation capacity of MDA-MB-231-Hyg-Zeb1-KO cells was markedly but not significantly lower compared to that of MDA-MB-231-Hyg cells (17 ± 2 colonies for MDA-MB-231-Hyg vs. 9 ± 2 colonies for MDA-MB-231-Hyg-Zeb1-KO; Figure 4A). In contrast, M13-MDA231-6-Zeb1-KO hybrids (56 ± 4 colonies; *p* < 0.0001) possessed a significantly higher colony formation capacity than M13-MDA231-6 hybrids (27 ± 4 colonies; Figure 4A), while that of M13-MDA231-13 hybrids and M13-MDA231-13-Zeb1-KO hybrids was rather similar (50 ± 2 colonies for M13-MDA231-13 vs. 51 ± 2 colonies for M13-MDA231-13-Zeb1-KO; Figure 4A). It is unclear what causes these differences.

### 2.5. The Migratory Activity of Zeb1-KO Cells Is Significantly Decreased in a Transwell/Boyden Chamber Assay

**Figure 5:**
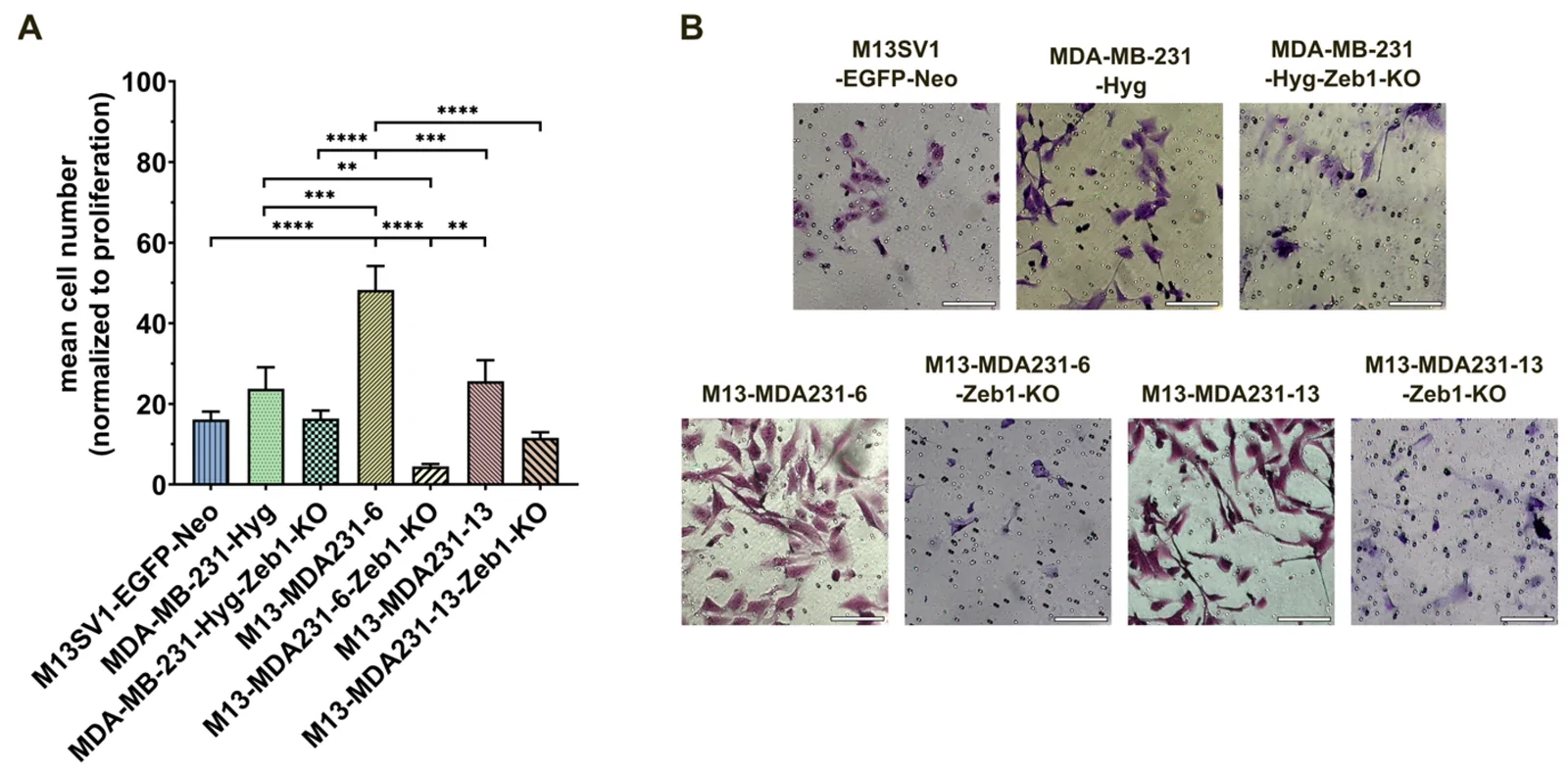
Zeb1-KO is associated with significantly decreased migratory activity. (**A**) The mean ± S.E.M. of at least three independent experiments. The number of transmigrated cells was normalized to the respective cell proliferation rate. Statistical significance was calculated using an ordinary one-way ANOVA and Tukey’s post hoc test. ** = *p* < 0.01; *** = *p* < 0.001; and **** = *p* < 0.0001. (**B**) Representative images of transmigrated cells. Bar = 100 µm.

As Zeb1 is important for the EMT process and, thus, for a more motile and invasive phenotype [31,33], we assumed that Zeb1-KO might be correlated with the cells’ decreased migratory activity. Therefore, the migratory activity of Zeb1-KO cells in comparison to their non-edited variants was analyzed using a Transwell/Boyden Chamber assay (Figure 5). Since the number of migrating cells can also be influenced by cell proliferation, the migration data were normalized based on the cell proliferation data.

Indeed, Zeb1-KO cells possessed a lower migratory activity compared to their non-edited variants, which was only significant for M13-MDA231-6-Zeb1-KO cells. Here, the migratory activity was virtually completely abrogated after Zeb1-KO (48 ± 6 cells for M13-MDA231-6 vs. 5 ± 1 cells for M13-MDA231-6-Zeb-KO; *p* < 0.0001; Figure 5A). In contrast, the migratory activity of MDA-MB-231-Hyg-Zeb1-KO cells (16 ± 2 cells) was moderately decreased compared to that of MDA-MB-231-Hyg cells (24 ± 5 cells; Figure 5A).

### 2.6. Zeb1-KO Cells Possess Markedly Lower Invasive Capacity

**Figure 6:**
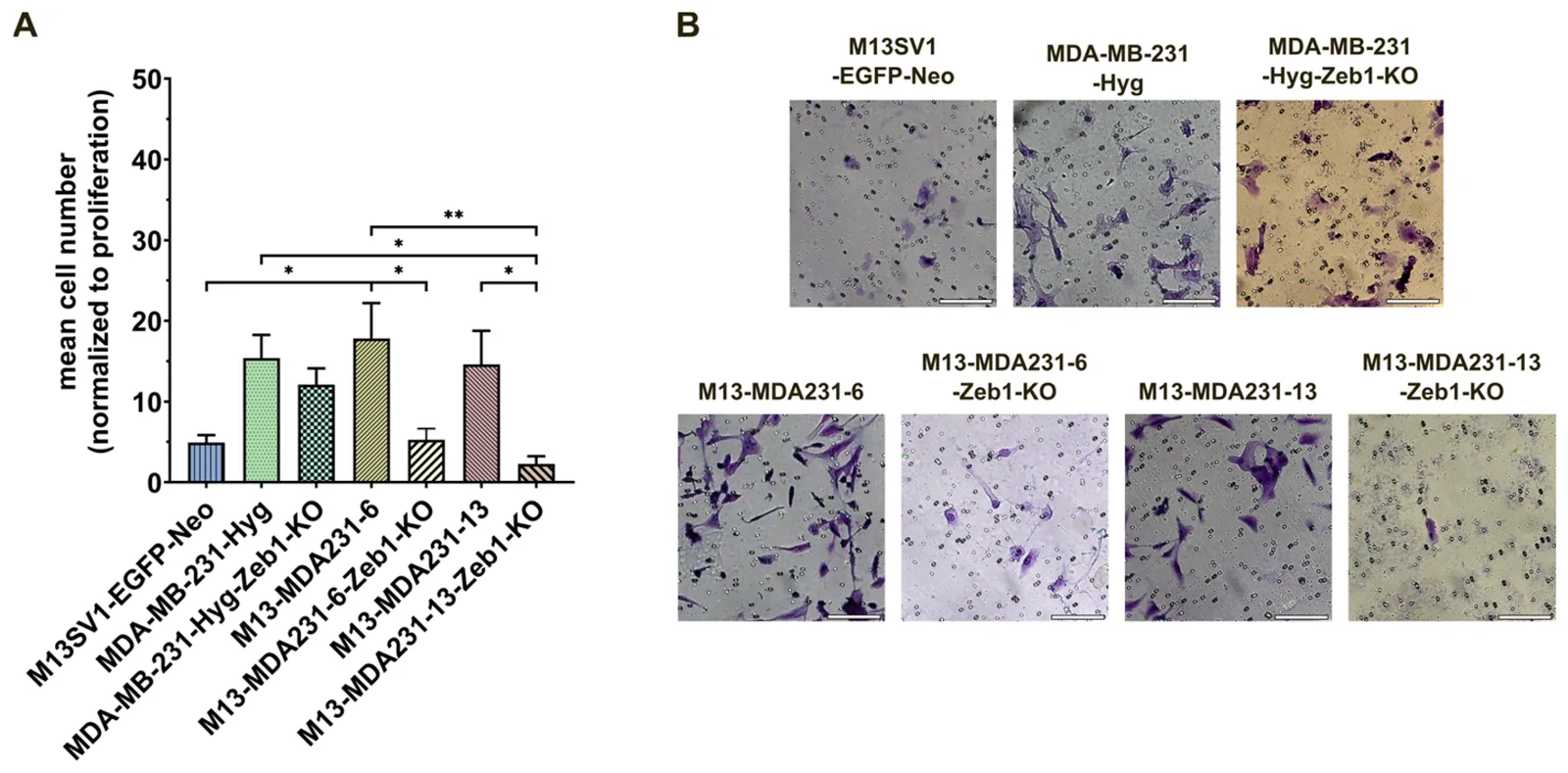
Zeb1-KO is associated with markedly decreased invasion capacity. (**A**) The mean ± S.E.M. of at least three independent experiments. The number of invaded cells was normalized to the respective cell proliferation rate. Statistical significance was calculated using an ordinary one-way ANOVA and Tukey’s post hoc test. * = *p* < 0.05; ** = *p* < 0.01. (**B**) Representative images of transmigrated cells. Bar = 100 µm.

In addition to the Transwell/Boyden Chamber assay, we also investigated the cells’ invasive capacity. Since the number of invaded cells can also be influenced by cell proliferation, the invasion data were normalized based on the cell proliferation data. The results are summarized in Figure 6 and clearly show that all Zeb1-KO cells possessed markedly lower invasion capacity.

The overall invasion capacity of M13SV1-EGFP-Neo was rather low (5 ± 1 cells; Figure 6), which is consistent with previous studies [29,34]. As expected, the invasive capacities of MDA-MB-231-Hyg and both M13-MDA231 hybrids were markedly higher. Interestingly, all cells showed a similar invasion capacity (MDA-MB-231-Hyg: 15 ± 3 cells; M13-MDA231-6: 18 ± 4 cells; M13-MDA231-13: 15 ± 4 cells; Figure 6A). However, Zeb1-KO was associated with a marked decrease in invasive capacity, although this was much more pronounced (and statistically significant) in the M13-MDA231 hybrids (M13-MDA231-6-Zeb1-KO: 5 ± 1 cells, *p* < 0.05; M13-MDA231-13-Zeb1-KO: 2 ± 1 cells, *p* < 0.05) (Figure 6A) than in the MDA-MB-231 hybrid cells (12 ± 2 cells; Figure 6A).

### 2.7. The Scratch/Wound Healing Assay Indicates Different Migratory Properties of Zeb1-KO Cells

**Figure 7:**
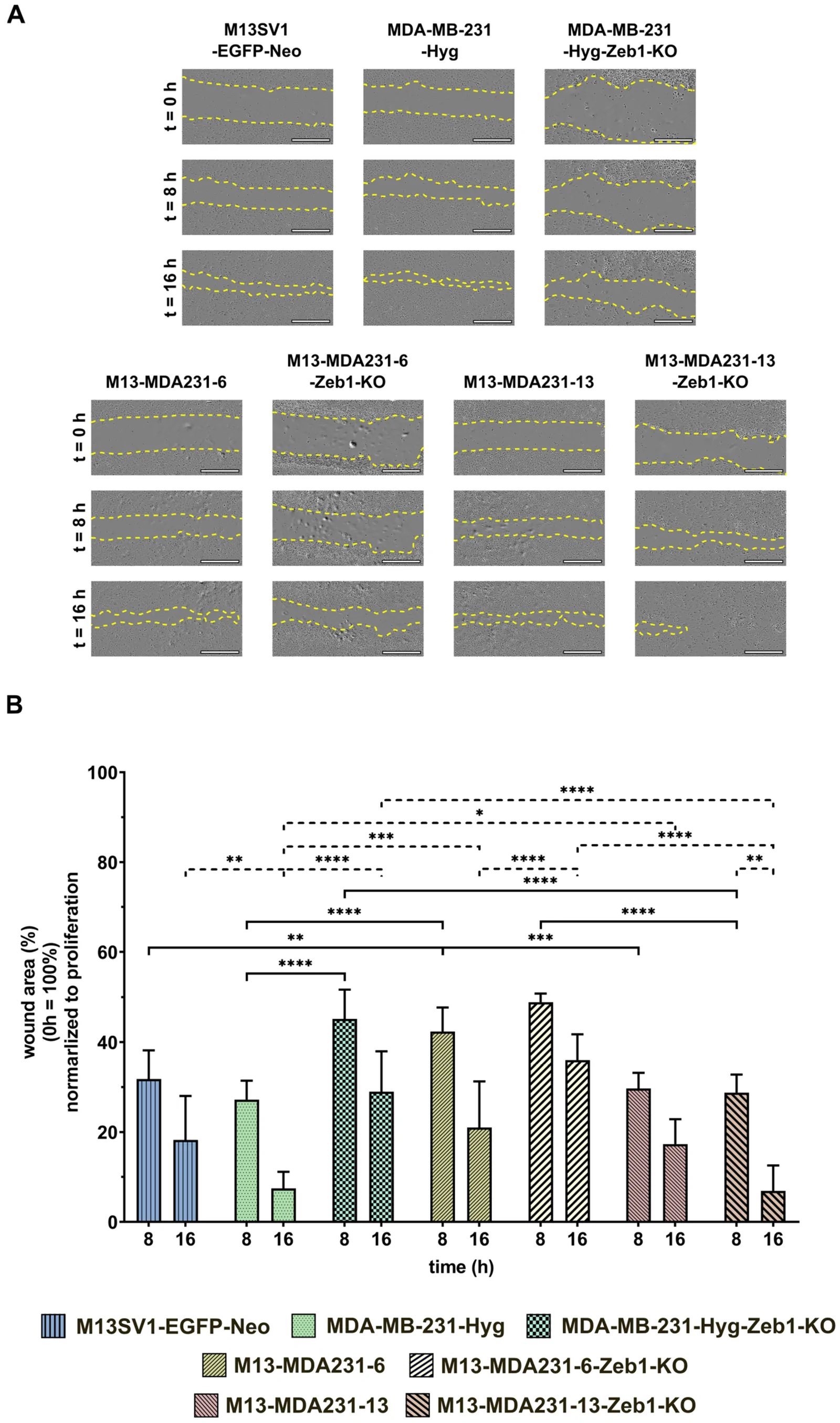
The scratch/wound healing assay indicates different migratory properties of Zeb1-KO cells. (**A**) Representative images at t = 8h and 16 h. The scratch/wound area is marked by a dashed yellow line. Bar = 1 mm. (**B**) Mean ± S.E.M. of the wound area of three independent experiments. The wound area was normalized to the respective cell proliferation rate. Statistical significance was calculated using a Two-Way ANOVA and Tukey’s post hoc test. * = *p* < 0.05; ** = *p* < 0.01; *** = *p* < 0.001; and **** = *p* < 0.0001. The bold lines indicate the statistical significance of the 8 h values, while the dashed lines show the statistical significance of the 16 h values.

In addition to Transwell/Boyden Chamber migration assay, we also performed a scratch/wound healing assay to study the cells’ migratory activity. Since the number of migrated cells can also be influenced by cell proliferation, the wound area was normalized based on the cell proliferation data. The results are summarized in Figure 7.

The scratch/wound healing migration data for M13SV1-EGFP-Neo, MDA-MB-231-Hyg, and MDA-MB-231-Hyg-Zeb1-KO cells (Figure 7B) were consistent with the Transwell/Boyden Chamber results (Figure 5A). MDA-MB-231-Hyg cells (mean wound area at 8h: 27 ± 1%; at 16h: 7 ± 1 (*p* < 0.01); Figure 7B) exhibited increased wound closure activity compared to M13SV1-EGFP-Neo cells (mean wound area at 8h: 32 ± 2%; at 16h: 18 ± 3%), while the migratory capacity of MDA-MB-231-Hyg-Zeb1-KO cells (mean wound area at 8h: 46 ± 3% (*p* < 0.0001); at 16h: 29 ± 4% (*p* < 0.0001); Figure 7B) was significantly lower compared to that of non-edited MDA-MB-231-Hyg cells. In accordance with transmigration data, M13-MDA231-6-Zeb1-KO cells also showed significantly reduced migratory activity (mean wound area at 8h: 49 ± 1%; at 16h: 36 ± 2% (*p* < 0.0001); Figure 7B) compared to M13-MDA231-6 hybrids (mean wound area at 8h: 42 ± 2%; at 16h: 21 ± 3%; Figure 7B). Interestingly, and in contrast to the results of the transmigration assay, the mean wound areas of the M13-MDA231-13-Zeb1-KO cells were smaller than those of the M13-MDA231-13 tumor hybrids (Figure 7B). The reason for this remains unclear.

### 2.8. Zeb1-KO Cells Exhibit Lower miR-200c-3p Levels than Non-Edited Parental Cells

**Figure 8:**
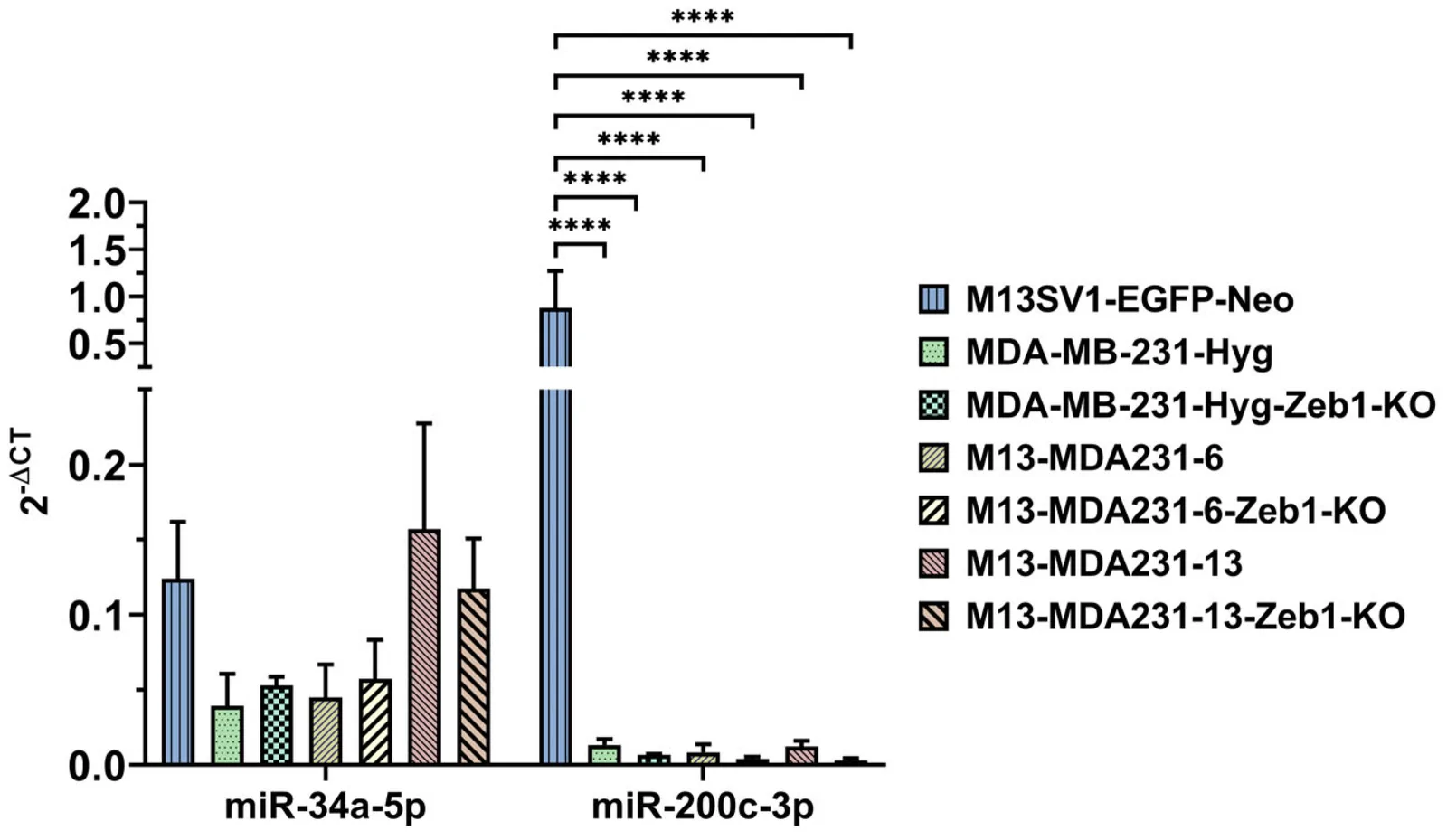
miR-34a-5p and miR-200c-3p expression patterns in non-edited cells and Zeb1-KO cells. The mean ± S.E.M of three independent experiments are shown. Statistical significance was calculated using an ordinary one-way ANOVA and Tukey’s post hoc test. **** = *p* < 0.0001.

Two EMT core networks have been suggested: “miR-200c-3p-Zeb1” and “miR-34a-5p-Snail” [3,5]. In this regard, it is well known that Zeb1 expression represses miR-200c-3p expression and vice versa [3,5]. Consistent with this, miR-34a-5p represses Snail expression and vice versa [3,5]. The miR-34a-5p expression data did not match with the cells’ Snail expression levels, which applies to both non-edited cells and Zeb1-KO cells. For example, M13SV1-EGFP-Neo expressed rather high levels of miR-34-5p compared to MDA-MB-231-Hyg cells (Figure 8) that did not fit to the cells’ Snail expression levels. Figure 2 shows that M13SV1-EGFP-Neo cells expressed markedly higher Snail levels than MDA-MB-231-Hyg cells. Similarly, MDA-MB-231-Hyg-Zeb1-KO cells exhibited markedly higher Snail expression levels than MDA-MB-231-Hyg cells (Figure 2) and also possessed slightly higher miR-34a-5p expression levels (Figure 8). Furthermore, M13-MDA231-13 hybrids showed the highest miR-34a-5p levels of all cells (Figure 8) regardless of Snail expression (Figure 2). In contrast, lower miR-34a-5p levels and Snail expression were observed in M13-MDA231-13-Zeb1-KO hybrids (Figure 2 and Figure 8).

In contrast to the miR-34a-5p data, the miR-200c-3p data of the non-edited cells matched well with Zeb1 protein expression (Figure 2 and Figure 8). Due to its epithelial phenotype and the lack of Zeb1 expression, high levels of miR-200c-3p were observed in M13SV1-EGFP-Neo cells (Figure 2 and Figure 8). Consistent with this, MDA-MB-231-Hyg cells and both M13MDA231 hybrids were positive for Zeb1 expression but virtually lacked miR-200c-3p expression (Figure 2 and Figure 8). Interestingly, qPCR data revealed that Zeb1-KO cells possessed even lower miR-200c-3p expression levels than their non-edited counterparts (Figure 8). This finding was rather unexpected due to the miR-200c-3p-Zeb1 EMT core network. Therefore, Zeb1-KO should have rather resulted in increased miR-200c-3p levels.

### 2.9. Effect of Zeb1-KO on the CD44/104 Expression Pattern

**Figure 9:**
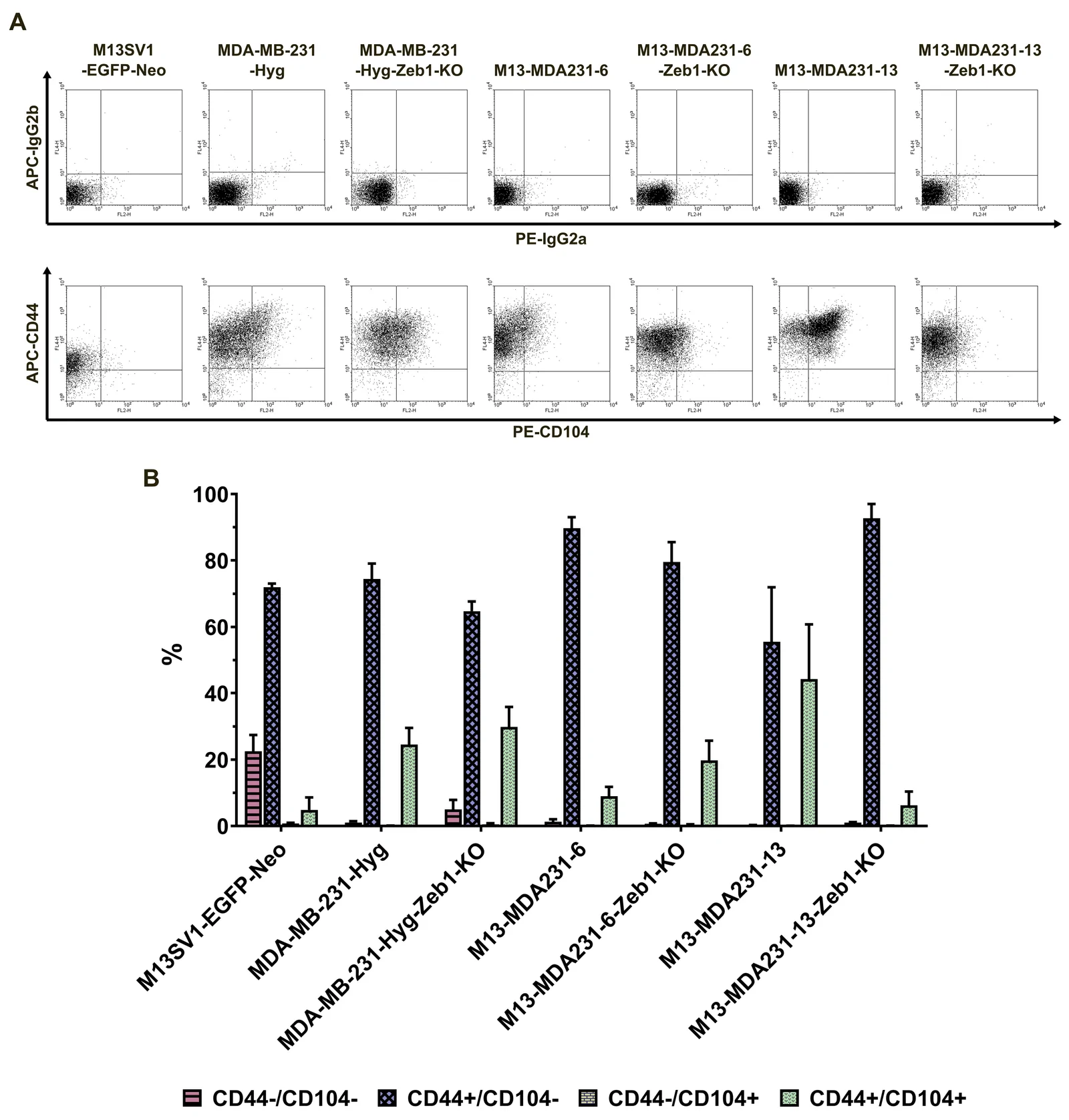
CD44+/CD104+ expression pattern in non-edited cells and Zeb1-KO cells. (**A**) Representative dot plots (isotype controls and CD44/CD104) of three independent experiments. (**B**) CD44/CD104 expression pattern. The mean ± S.E.M. of three independent experiments are shown. Statistical analysis was calculated using a Two-Way ANOVA and Tukey’s post hoc test and revealed no statistical significance.

The membrane-bound molecules CD44 and CD104 have been identified as suitable markers for the E, hybrid E/M, and M states of breast cancer cells [9,10]. Flow cytometry data are summarized in Figure 9 and clearly show marked differences in the amount of CD44+/CD104+ cells. For example, about 24.5 ± 5.0% of MDA-MB-231-Hyg cells, 8.9 ± 2.9% of M13-MDA231-6 hybrids, and 44.3 ± 16.5% of M13-MDA231-13 hybrids were CD44+/CD104+ (Figure 9B). These results also indicate that both hybrid clones differed markedly in the frequency of CD44+/CD104+ cells. The Zeb1-KO in MDA-MB-231-Hyg and M13-MDA231-6 hybrids was correlated with increased numbers of CD44+/CD104+ cells (MDA-MB-231-Hyg-Zeb1-KO: 29.8 ± 5.0%; M13-MDA231-6-Zeb1-KO: 19.7 ± 5.9%; Figure 9). Interestingly, the Zeb1-KO in M13-MDA231-13 hybrids was associated with a marked reduction in the number of CD44+/CD104+ cells to only 6.2 ± 4.2% (Figure 9).

### 2.10. The Cellular Morphology Is Altered in MDA-MB-231-Hyg-Zeb1-KO Cells but Not in M13-MDA231-Zeb1-KO Hybrid Clones

**Figure 10:**
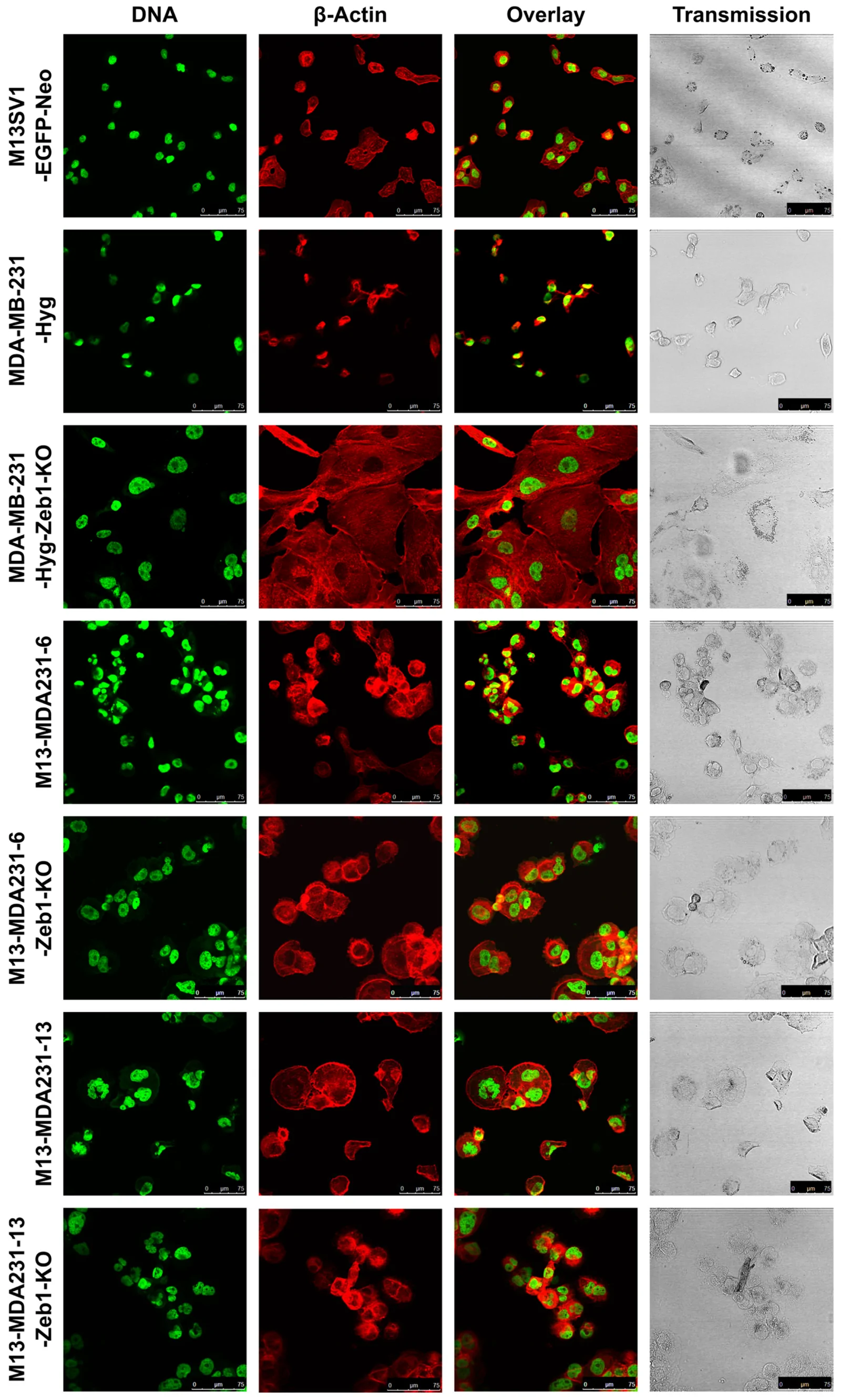
The morphology of MDA-MB-231-Hyg cells, but not M13-MDA231 hybrid clones, is markedly changed after Zeb1-KO. All cells were seeded in chamberslides and then fixed and stained. DNA was stained with Sytox^®^ Green, while the actin cytoskeleton was stained with Alexa Fluor^®^ 568-phalloidin. Images were acquired using confocal laser scanning microscopy. Representative images of two independent measurements are shown. Bar = 75 µm.

Finally, the cells were characterized by confocal laser scanning microscopy to investigate whether Zeb1-KO was associated with an altered morphology. The results are summarized in Figure 10. Due to its epithelial phenotype, M13SV1-EGFP-Neo cells possessed a round shape morphology, while the morphology of MDA-MB-231-Hyg cells was rather elongated and mesenchymal-like (Figure. 10). Interestingly, the morphology of MDA-MB-231-Hyg-Zeb1-KO was markedly different. While some cells still displayed a more mesenchymal-like phenotype, MDA-MB-231-Hyg-Zeb1-KO cells were markedly larger and more round-shaped than their non-edited counterparts (Figure 10). In contrast, the morphology of M13-MDA231-hybrid clones was more round-shaped and epithelial-like (Figure 10). Notably, the morphology of M13-MDA231-Zeb1-KO hybrid clones was not altered compared to their non-edited counterparts (Figure 10).

## 3. Discussion

In the present study, we investigated CRISPR/Cas9-mediated KO of Zeb1 in MDA-MB-231-Hyg human breast cancer cells and M13-MDA231 tumor hybrids, which were derived from spontaneous fusion events between MDA-MB-231-Hyg cells and M13SV1-EGFP-Neo human breast epithelial cells [28]. The rationale behind this study was that Zeb1 is a well-known EMT transcription factor [1,2,3,4].

Western blot results show that M13SV1-EGFP-Neo human breast epithelial cells exhibited a classical E state. The cells expressed E-Cadherin and Cytokeratin 5, which is consistent with previous data [29,34]. Interestingly, in accordance with previous results [29,34], M13SV1-EGFP-Neo cells also expressed Snail. The reason for this finding remains unclear, as Snail is a rather well-known EMT transcription factor [32]. Therefore, the finding that M13SV1-EGFP-Neo cells are positive for Snail expression is most likely attributed to cell line-specific characteristics. In contrast, MDA-MB-231-Hyg cells and M13-MDA231 tumor hybrids exhibit a more mesenchymal-like phenotype as they all express Zeb1 and Vimentin. Interestingly, our results indicate that MDA-MB-231-Hyg did not express N-Cadherin. When comparing studies in the literature, the results for MDA-MB-231 cells and N-Cadherin expression were different. While some studies showed that MDA-MB-231 cells expressed N-Cadherin (for example, [35,36,37]), other works demonstrated that MDA-MB-231 cells were clearly negative for N-Cadherin expression (for example, [38,39,40]). Moreover, even “The Human Protein Altas” stated that N-Cadherin expression was not detectable by mass spectroscopy in the MDA-MB-231 cell line (https://www.proteinatlas.org/ENSG00000170558-CDH2/cell+line; accessed on 15 June 2026). The reasons for these differences remain unknown. Since we regularly verify the authenticity of the cell lines and have not yet observed any abnormalities in the STR profile, we assume that the cell line used in this study comprised MDA-MB-231 cells.

Notably, MDA-MB-231-Hyg and both M13-MDA231 tumor hybrids expressed Snail. Given that Snail and Zeb1 are determinants of the mixed E/M state that is associated with stemness traits, it may be speculated that these cells possess CSC characteristics. However, our previous results show that both the fraction of ALDH+ cells and the overall mammosphere formation capacity of MDA-MB-231-Hyg and M13-MDA231-6 and -13 tumor hybrids was rather low [28]. We only observed increased colony formation capacity of M13-MDA231 tumor hybrids [28], which is consistent with the data presented in this study.

The finding that the Zeb1-KO in MDA-MB-231-Hyg breast cancer cells was associated with re-induction of E-Cadherin expression is consistent with published data [41,42,43]. For example, RNAi-mediated knock-down of Zeb1 reactivated E-Cadherin expression in MDA-MB-231 breast cancer cells [41]. Consistent results were obtained using a Zeb1-specific shRNA approach [42]. Interestingly, the shRNA-mediated knock-down of Zeb1 expression was also associated with reduced protein expression levels of Vimentin [42], which was not the case in this study, where all Zeb1-KO and non-edited cells showed comparable protein expression levels of Vimentin. Nagai et al. overexpressed miR-200c-141, a member of the miR-200c family, to successfully reduce the expression of Zeb1 in MDA-MB-231 cells [43]. Notably, Vimentin expression was not diminished in these cells [43], which is consistent with our results. Interestingly, Zeb1-KO was associated with markedly higher expression levels of the EMT transcription factor Snail in MDA-MB-231-Hyg cells. This result was rather unexpected, as Snail is a known repressor of E-cadherin in epithelial tumors [44,45]. However, as shown in this study, MDA-MB-231-Hyg-Zeb1-KO cells expressed E-cadherin. In this regard, it is also unclear why the Zeb1-KO in M13-MDA231 tumor hybrids was associated with reduced Snail expression but increased N-cadherin expression. It is well known that the expression of N-Cadherin is induced by the EMT transcription factor Snail [45,46]. The underlying cause of these differences is unknown. It cannot be ruled out that other EMT transcription factors were involved in the up-regulation of E-Cadherin in MDA-MB-231-Hyg-Zeb1-KO cells or of N-cadherin in M13-MDA-231-Zeb1-KO tumor hybrids.

The overall effects of the Zeb1-KO in MDA-MB-231-Hyg and M13-MDA231 tumor hybrid clones are difficult to interpret. Both cell proliferation and the ability to form colonies were significantly lower in the MDA-MB-231-Hyg-Zeb1-KO cells compared to the non-edited cells. These results are in accordance with the known role of Zeb1 in promoting cell proliferation and stemness [47,48]. However, in M13-MDA231-6 tumor hybrids, Zeb1-KO was associated with significantly increased cell proliferation rate and colony formation capacity. Similarly, proliferation of M13-MDA231-13-Zeb1-KO tumor hybrids was significantly decreased, while the cells’ colony formation capacity was comparable to that of non-edited M13-MDA231-13 cells. Therefore, the effects of the Zeb1-KO in M13-MDA231 tumor hybrid clones were rather contrary to the described role of Zeb1 in promoting cell proliferation. It is unclear why the Zeb1-KO cells exhibited such different colony-forming behavior compared to the unedited cells. We assume that (hybrid) cell line-specific differences are responsible for this. It is known that the ability of potential cancer stem cells to form colonies depends on several stemness factors, such as Oct4 and Sox2, as well as signaling pathways, including the Wnt and Notch signaling cascades [49]. Without knowing which stemness factors are expressed by the non-edited cells and the Zeb1-KO cells, and which signaling pathways are active, no conclusions can be drawn as to what causes these differences. This also applies to the CD44/CD104 flow cytometry data. CD44/CD104 expression has been suggested as a marker pattern to distinguish between breast cancer cells that are either in an E, M, or mixed E/M state [9,10]. Thereby, CD44+/CD104+ cells belong to the mixed E/M state, further exhibiting CSC properties [9,10]. Flow cytometry results show that the frequency of CD44+/CD104+ cells was increased in MDA-MB-231-Hyg-Zeb1-KO and M13-MDA231-6-Zeb1 cells, which does not match the cells’ decreased capacity to from colonies. In contrast, M13-MDA231-13-Zeb1-KO tumor hybrids harbored a markedly lower population of CD44+/CD104+ cells, while the colony formation capacity was rather identical to that of non-edited cells. However, it must be noted that neither the ability to form colonies nor the expression pattern of CD44+/CD104+ on the cells allows us to conclude whether the cells possess potential cancer stem cell properties. This also applies to the mammosphere formation [50] and ALDH1+ assays [51], which are additional in vitro assays used to screen cancer cells for potential stemness properties. Ultimately, however, only xenograft limited-dilution assays will provide reliable results in this regard [52,53]. Therefore, animal experiments are recommended to investigate the tumor-inducing properties of the cells and how they are affected by Zeb1-KO.

Transmigration and cell invasion results show that the transmigratory and invasive capacities of Zeb1-KO cells were significantly reduced compared to those of non-edited cells. These findings are consistent with the role of Zeb1 in EMT and the promotion of a more motile and invasive phenotype [47,54]. Furthermore, our results are consistent with published data [54,55,56,57,58]. For example, the siRNA-mediated knock-down of Zeb1 partially restored the epithelial phenotype and reduced transendothelial migration of prostate cancer cells [54]. Similarly, expression of miR-200 members in MDA-MB-231 breast cancer cells and 4TO7 murine mammary carcinoma cells was associated with loss of Zeb1 expression, reinduction of E-Cadherin expression, and markedly decreased migratory and invasive capacities [55,56,58]. We also observed decreased migratory activity of MDA-MB-231-Hyg-Zeb1-KO and M13-MDA231-6-Zeb1-KO in the scratch/wound healing assay, but not for M13-MDA231-13-Zeb1-KO tumor hybrids. Interestingly, these cells exhibited higher locomotory activity. On the one hand, it must be noted that the scratch/wound healing assay and the Transwell/Boyden Chamber are only partially comparable. Although both assays are commonly used to study the migratory behavior of cells, the read-out is different. A Transwell/Boyden Chamber only quantifies cells that have passed through the membrane into the lower compartment [59]. In contrast, in a scratch/wound healing assay, the size of the scratch/wound is used for quantification, which is related to the number of cells that have moved into the scratch/wound [60]. It may also be speculated that the differences in migration behavior between M13-MDA231-13 and M13-MDA231-13-Zeb1-KO cells, compared to other cell lines, could be attributed to different proliferation patterns. However, this is rather unlikely since the respective proliferation rates have already been taken into account in the migration data. Furthermore, M13-MDA231-6-Zeb1-KO and M13-MDA231-13-Zeb1-KO cells exhibited nearly identical proliferation rates but differed in their migration behavior in the scratch/wound healing assay. Therefore, the increased migration of M13-MDA231-Zeb1-KO cells in the scratch/wound healing assay is most likely attributed to other mechanisms.

As mentioned above, the miR-200c-3p-Zeb1 regulatory feedback loop is a crucial part of the core EMT regulatory networks [3,5]. In accordance with previous data [29], we also observed that Zeb1-KO was not correlated with increased miR-200c-3p levels. In fact, these results are in contrast to data obtained by Sundarayan et al., who showed that MDA-MB-231-shRNA Zeb1 cells exhibited significantly higher expression levels of miR-200c [56]. However, miR-200c-3p belongs to the miR-200 family that consists of five members: miR-200a, miR-200b, miR-200c, miR-141, and miR-429 [58,61]. Indeed, expression of miR-141 in MDA-MB-231 breast cancer cells was associated with decreased Zeb1 expression levels and re-induction of E-Cadherin expression [55]. Similarly, transfection of HEY ovarian cancer cells with miR-429 also resulted in reduced expression levels of Zeb1 [62]. Notably, siRNA-mediated knock-down of Zeb1 in HEY cells was correlated with increased miR-429 levels, which supports the miR-429-Zeb1 negative feedback loop [62]. In this regard, it is worth investigating what effect Zeb1-KO might have on the expression of other miR-200 family members. The theory that other miR-200 family members might be involved in the regulation of Zeb1 in MDA-MB-231 and M13-MDA231 tumor hybrids cannot be ruled out, even though the data presented by Sundarayan et al. tend to suggest otherwise [56]. This is further supported by the fact that miR-200c-3p was highly expressed in M13SV1-EGFP-Neo breast epithelial cells, which would support the miR-200c-3p-Zeb1 negative feedback loop.

## 4. Materials and Methods

### 4.1. Cell Culture

M13SV1-EGFP-Neo, MDA-MB-231-Hyg, M13-MDA231-6, and M13-MDA231-13 cells were generated and cultivated as described [28]. Briefly, human M13SV1-EGFP-Neo cells breast epithelial cells were derived from M13SV1 cells by stable transfection with the pEGFP-MCS-Neo plasmid [27]. M13SV1 cells were kindly provided by James Trosko (Michigan State University, East Lansing, MI, USA [63]). M13SV1-EGFP-Neo cells were maintained in RPMI 1640 media (PAN Biotech GmbH, Aidenbach, Germany) supplemented with 10% fetal bovine serum (PAN Biotech GmbH, Aidenbach, Germany), 100 U/mL penicillin/0.1 mg/mL streptomycin (PAN Biotech GmbH, Aidenbach, Germany), 0.5 ng/mL of recombinant human epidermal growth factor, 5 µg/mL of human recombinant insulin, 0.5 µg/mL of hydrocortisone, 4 µg/mL of human transferrin, 10 nM of β-estrogen, and 400 µg/mL of G418 (all supplements were purchased from Merck KGaA, Darmstadt, Germany). MDA-MB-231-Hyg human breast cancer cells were derived from MDA-MB-231 cells (HTB 26; LGC Standards GmbH, Wesel, Germany) via stable transfection with the pKS-Hyg plasmid [28] and were cultured in DMEM media (PAN Biotech GmbH, Aidenbach, Germany) supplemented with 10% fetal bovine serum (PAN Biotech GmbH, Aidenbach, Germany), 100 U/mL penicillin/0.1 mg/mL streptomycin (PAN Biotech GmbH, Aidenbach, Germany), and 200 µg/mL of Hygromycin B (Pan Biotech, Aidenbach, Germany). M13-MDA231-6 and M13-MDA231-13 hybrid cells were derived from spontaneous fusion events between M13SV1-EGFP-Neo cells and MDA-MB-231-Hyg cells [28]. M13-MDA231 hybrids were cultured in DMEM media (PAN Biotech GmbH, Aidenbach, Germany) supplemented with 10% fetal bovine serum (PAN Biotech GmbH, Aidenbach, Germany), 100 U/mL penicillin/0.1 mg/mL streptomycin (PAN Biotech GmbH, Aidenbach, Germany), 200 µg/mL of Hygromycin B (Pan Biotech, Aidenbach, Germany) and 400 µg/mL of G418 (Merck KgaA, Darmstadt, Germany). All cells were cultured at 37 °C and 5% CO_2_ in a humidified atmosphere.

### 4.2. Generation of Zeb1-Knockout (KO) Cells

CRISPR/Cas9-mediated Zebl-KO variants of MDA-MB-231-Hyg, M13-MDA231-6, and M13-MDA231-13 cells were generated as described recently [29]. The Zeb1-specific guide RNA (5′-GAG CAC TTA AGA ATT CAC AG-3′) was adopted from the study of Kroger and colleagues [10]. Sense and antisense oligonucleotides (gRNA_ZEB1_fwd: 5′-CAC CGA GCA CTT AAG AAT TCA CAG-3′, gRNA_ZEB1_rev: 5′-AAA CCT GTG AAT TCT TAA GTG CTC-3′; Thermo Fisher Scientific, Wesel, Germany) were annealed and ligated into the BbSI digested pX330-U6-Chimeric_BB-CBh-hSpCas9-P2A-PuroR vector-plasmid as described in [29]. The pX330-U6-Chimeric_BB-CBh-hSpCas9-P2A-PuroR plasmid was generated by inserting a FseI-p2A-PuroR-bGH Poly(A)-NotI fragment from pcDNA3.1_iCre-T2A-mCherry-p2A-PuroR into a Fse1/NotI restricted pX330-U6-Chimeric_BB-CBh-hSpCas9 plasmid (pX330-U6-Chimeric_BB-CBh-hSpCas9 was a gift from Feng Zhang; Addgene plasmid #42230; http://n2t.net/addgene:42230; accessed on 8 July 2026; RRID: Addgene_42230) [29,34]. Successful cloning was verified by Sanger sequencing (Eurofins Genomics, Ebersbach, Germany). The CRISPR2/Cas9-sgZeb1 plasmid was amplified in NEB 10β bacteria (New England Biolabs GmbH, Frankfurt am Main, Germany) and purified using the Nucleospin^®^ Plasmid Transfection-grade in accordance with the instruction manual (Macherey-Nagel GmbH, Düren, Germany).

Cells (MDA-MB-231-Hyg, M13-MDA231-6, and M13-MDA231-13) were transiently transfected with CRISPR2/Cas9-sgZeb1 plasmid (1–2 µg) using the jetOPTIMUS^®^ DNA transfection reagent (Polyplus, Illkirch, France) according to the manufacturer’s protocol. Non-transfected cells were removed by Puromycin selection. Therefore, after transfection for 72h, Puromycin (2 µg/mL; Thermo Fisher Scientific, Wesel, Germany) was added to the culture medium and left for 24h. Surviving cells were maintained until single-cell-derived colonies with an average diameter of 2–3 mm were formed. Colonies were picked by using 3 mm Whatman paper disks soaked with Trypsin 0.25%/1 mM of EDTA (PAN Biotech GmbH, Aidenbach, Germany) and then transferred to 96-well plates (Sarstedt AG & Co. KG, Nümbrecht, Germany) for further propagation.

Successful CRISPR/Cas9 editing of the Zeb1 gene was verified by Sanger sequencing (Eurofins Genomics, Ebersbach, Germany). Therefore, genomic DNA was isolated from Zeb1-KO cells using the NucleoSpin^®^ Tissue DNA kit according to the instruction manual (Macherey & Nagel, Düren, Germany). First, the CRISPR/Cas9 edited gene sequence was amplified by PCR (PCR_ZEB1-fwd. 5′-TCC TGT CTT CTA TTC AGG ACC-3′; PCR_ZEB1 rev. 5′-GAA CTT GTT TTC GCG TTT TCC-3′; Thermo Fisher Scientific, Wesel, Germany). Subsequently, the PCR product was subjected to Sanger sequencing (Eurofins Genomics, Ebersbach, Germany) using the sequencing primers (Seq_ZEB1_7_fwd. 5′-GGA AAG CAA ACA AGT TAA CCT C-3′ and Seq_ZEB1_7_rev. 5′-TGT AAT CCT TTC ACT CCC TCT C-3′; Thermo Fisher Scientific, Wesel, Germany). Obtained sequences were analyzed with SnapGene 5.3.1 software (Dotmatics, Bishops Stortford, UK). Successfully edited CRISPR/Cas9 Zeb1-KO clones of MDA-MB-231-Hyg, M13-MDA231-6, and M13-MDA231-13 cells were pooled and expanded for further studies.

### 4.3. Western Blot Analysis

Western blot analysis of target genes was performed using total cell lysates. Therefore, 20 µL of cell suspension (2 × 10^5^ cells/20 µL PBS) was combined with 10 µL of 3× Laemmli Sample Buffer and incubated for 10 min at 95 °C. Samples were separated using either 10% or 12% sodium dodecylsulfate–polyacrylamide gel electrophoresis (SDS-PAGE) and then transferred to an Immobilon polyvinyldifluoride (PVDF) membrane (Merck Millipore, Darmstadt, Germany) or an Amersham^TM^ Protran^TM^ 0.45 µm NC nitrocellulose blotting membrane (Merck KGaA, Darmstadt, Germany) under semi-dry conditions. As recommended by the manufacturers of the used primary and secondary antibodies, membranes were either blocked with 5% (*w*/*v*) non-fat milk powder or 5% bovine serum albumin (BSA) in Tris-buffered saline with 1% (*v*/*v*) Tween 20 (TBS-T). The Pierce ECL Western Blot substrate (Thermo Fisher Scientific, Wesel, Germany) and the Aequoria Macroscopic Imaging System (Hamamatsu Photonics Germany, Herrsching am Ammersee, Germany) was used for visualization of the bands. The following antibodies were used in this study: β-actin, E-Cadherin, Cytokeratin-5, N-cadherin, Snail, Vimentin, Zeb1, anti-mouse IgG-HRP-linked, and anti-rabbit IgG-HRP-linked. The β-Actin antibody was purchased from Merck KGaA, Darmstadt, Germany. All other antibodies were obtained from Cell Signaling Technology Europe B.V., Frankfurt am Main, Germany. Catalog numbers, clone numbers, and dilution are summarized in Table S1.

### 4.4. Colony Formation Assay

The colony formation assay was performed as described previously [28,29]. In brief, cells were seeded in 6-well plates (200 cells/well) and cultured for 14 days in a humidified atmosphere at 37 °C and 5% CO_2_. Subsequently, the media was removed and the cells were washed twice with PBS and then fixed with 4% paraformaldehyde solution (Agilent Technologies Deutschland GmbH, Waldbronn, Germany) for 15 min at RT. Then, the fixed colonies were stained with 0.5% crystal violet solution (Merck KGaA, Darmstadt, Germany) for 1 h at RT. Fixed samples were thoroughly washed twice with PBS and once with deionized water and then air-dried. The stained 6-well plates were scanned using a Sharp BP-55C26 MFP device (Sharp, Cologne, Germany) and analyzed using Fiji software (Image J 1.54f; https://Fiji.sc; accessed on 29 June 2023). Colonies were manually counted using the Cell Counter plugin of the Fiji software.

### 4.5. Transwell/Boyden Chamber Assay and Invasion Assay

The Transwell/Boyden Chamber migration and invasion assays were carried out as described previously [29,34]. For the Transwell/Boyden Chamber migration assay, cells (6.5 × 10^4^ cells) were resuspended carefully in serum-free media and pipetted into the upper compartment of the Transwell insert (diameter of 6.5 mm and 8 µm pore size; Becton Dickenson, Heidelberg, Germany), which then was placed in a 24-well plate (Sarstedt AG & Co KG, Nümbrecht, Germany). The lower compartment was filled with 250 µL of complete media.

For invasion studies, Transwell inserts were coated with 100 µL of a 4 °C cold Geltrex solution (1:4 diluted with 4 °C cold PBS; Thermo Fisher Scientific, Wesel, Germany). Then, the Transwell inserts were incubated for 60 min at 37 °C for polymerization. Subsequently, cells (6.5 × 10^4^ cells) were resuspended in serum-free media and pipetted carefully on top of the polymerized Geltrex matrix. The lower compartment was filled with 250 µL of complete media.

Both assays were cultivated for 24 h in a humidified atmosphere at 37 °C and 5% CO_2_. Then, the Transwell inserts were removed, and any remaining cells in the upper compartment were gently removed with a cotton swab. Then, transmigrated cells were fixed with 4% paraformaldehyde (Agilent Technologies Deutschland GmbH, Waldbronn, Germany) for 15 min at RT, washed twice with PBS, and stained with 1% crystal violet staining solution (Merck KGaA, Darmstadt, Germany) for 1 h at RT. Fixed samples were thoroughly washed twice with PBS and once with deionized water and then air-dried. Invaded/transmigrated cells were visualized using an inverted microscope (Leica DM IRB; Leica, Wetzlar, Germany) and the Zeiss Labscope software (version 4.3.0; Carl Zeiss Microscopy GmbH, Jena, Germany). Thereby, six randomly chosen fields were captured per Transwell insert. The number of invaded/transmigrated cells was quantified using the Cell Counter plugin of the Fiji software (Image J 1.54f; https://Fiji.sc; accessed on 29 June 2023). The number of transmigrated and invading cells was additionally normalized to the respective cell proliferation rate, using the fold change between 24 and 48 h.

### 4.6. Scratch/Wound Healing Assay

The scratch/wound healing assay was carried out as described previously [29]. Cells were seeded in triplicates (2–2.5 × 10^5^ cells/well) in a 24-well plate (Sarstedt AG & Co KG, Nümbrecht, Germany) and cultivated in a humidified atmosphere at 37 °C and 5% CO_2_ until they reached 100% confluency. The scratch/wound was created with a 10 µL pipette tip. Dead cells and cell debris were removed with one careful PBS washing step. Then, fresh media (1.5 mL) was applied to the cells, and the 24-well plate was placed in the Incucyte^®^ SX5 Live-Cell Imaging system (Sartorius Lab Instruments GmbH, Göttingen, Germany). Transmission light images were taken every four hours for a total of 24 h at 37 °C and 5% CO_2_. The Fiji software (Image J 1.54f; https://Fiji.sc; accessed on 29 June 2023) was applied to determine the scratch/wound closure, whereby the scratch/wound size at defined time points were calculated in relation to the scratch/wound size at t = 0 h, which was set to 100%. The determined wound size was additionally normalized to the respective cell proliferation rate, using the fold change between 24 and 48 h.

### 4.7. Flow Cytometry

The CD44 and CD104 expression levels were determined by flow cytometry using a FACSCalibur flow cytometer (Becton Dickenson, Heidelberg, Germany). Therefore, cells 2 × 10^5^/100 µL) were co-stained with either PE- and APC-matched isotype controls (PE mouse IgG2a κ; clone MOPS-173; BioLegend, Amsterdam, the Netherlands; APC mouse IgG2b κ; clone 27-35; Becton Dickenson, Heidelberg, Germany) or PE-CD104- and APC-CD44-specific antibodies (PE-CD104; clone 58XB4; BioLegend, Amsterdam, the Netherlands; APC-CD44; clone G44-26; Becton Dickenson, Heidelberg, Germany) for 30 min at 37 °C. Concentrations of isotypes and specific antibodies were used in accordance with the manufacturer’s guidelines. Stained cells were washed once in PBS before flow cytometry analysis. Flow cytometry data were analyzed using the WinMDI software version 2.8 (http://www.cyto.purdue.edu/flowcyt/software/Winmdi.htm; accessed on 1 February 2020).

### 4.8. qPCR

Total RNA was isolated from cells using the NucleoSpin^®^ RNA kit as described in the instruction manual (Macherey & Nagel, Düren, Germany). The amount and quality of purified RNA was analyzed by photometry using an Eppendorf D30 BioPhotometer (Eppendorf SE, Hamburg, Germany). An A_260_/A_280_ ratio of 1.9 to 2.1 was considered pure RNA. If not used immediately, RNA was stored at −80 °C. cDNA was prepared for miRNA analysis using the TaqMan^®^ advanced miRNA-cDNA synthesis kit in accordance with the manufacturer’s instructions (Thermo Fisher Scientific, Wesel, Germany). The relative expression of miR-200c-3p and miR-34a-5p in relation to let-7a-5p was determined using the following specific assays for miRNA quantification: hsa-miR-200c-3p (assay ID 002300), hsa-miR-34a-5p (assay ID 000426), and hsa-let-7a-5p (assay ID 000377; housekeeping miRNA). All miRNA assays were purchased from Thermo Fisher Scientific, Wesel, Germany). qPCR was run on QuantStudio 1 Real-Time-PCR-system using cDNA, miRNA assays, and the TagMan^®^ advanced master mix (qPCR cycler and all reagents were from Thermo Fisher Scientific, Wesel, Germany). Data were analyzed using the QuantStudio^TM^Design&Analysis software (v1.5.3; Thermo Fisher Scientific, Wesel, Germany). The relative expression levels of miR-200c-3p and miR-34a-5p were calculated in relation to the internal control hsa-let-7a-5p using the ΔCT method.

### 4.9. Confocal Laser Scanning Microscopy

The morphology of non-edited and Zeb1 edited cells was visualized by confocal laser scanning microscopy (Leica TCS SP5; Leica Microsystems, Wetzlar, Germany). Cells were seeded in chamberslides (2 × 10^4^ cells/well; Nunc Lab-Tek; Thermo Fisher Scientific, Wesel, Germany) for up to 48h in a humidified atmosphere at 37 °C and 5% CO_2_. Subsequently, the cells were fixed with 4% paraformaldehyde (Merck KGaA, Darmstadt, Germany) for 15 min at RT and then washed twice with PBS. Thereafter, cells were permeabilized with 1% Triton X-100 ((*v*/*v*) in PBS) for 5 min at RT and again washed twice with PBS. The DNA was stained with SYTOX^®^-Green (Thermo Fisher Scientific, Wesel, Germany) for 15 min at room temperature in the dark, whereas the Alexa Fluor^®^ 568-phalloidin (Thermo Fisher Scientific, Wesel, Germany) was used for staining of the actin cytoskeleton (30 min, RT). Finally, cells were thoroughly washed with PBS and mounted with Fluoromount (Thermo Fisher Scientific, Wesel, Germany).

### 4.10. Statistical Analysis

Statistical significance was calculated using the GraphPad PRISM software 8.4.3 (graphpad.com; https://www.graphpad.com, accessed on 1 February 2020). Data are presented as the mean ± standard error of the mean (S.E.M.). Parametric data were analyzed using a one-way analysis of variance (ANOVA) and Tukey’s multiple comparison post hoc test. Non-parametric data were analyzed using the Kruskal–Wallis test and Dunn’s multiple comparison post hoc test. The following *p*-values were considered significant: * = *p* < 0.05, ** = *p* < 0.01, *** = *p* < 0.001, and **** = *p* < 0.0001.

## 5. Conclusions

In conclusion, our data indicate that Zeb1 is associated with EMT in MDA-MB-231-Hyg and M13-MDA231 tumor hybrids. In fact, both migratory and invasive properties were diminished in Zeb1-KO cells, while data for proliferation, colony formation, and CD44/CD104 expression were rather inconsistent. The reason why individual M13-MDA231 tumor hybrids and their Zeb1-KO variants exhibited different characteristics regarding, e.g., proliferation, CD44/CD104 expression, and colony formation, is most likely attributed to the manifold genomic alterations that are associated with cell fusion. For example, parental chromosomes are first mixed and then randomly segregated to daughter cells—a process commonly associated with chromosome missegregation, lagging chromosomes, chromothripsis, and micronuclei formation [64,65]. Furthermore, the initial tumor hybrid karyotype is fine-tuned in subsequent cell divisions until the tumor hybrids reach a rather stable karyotype [64,65]. All of these processes run in evolving tumor hybrids in a random, unpredictable manner [64,65]. In this regard, it is interesting that tumor hybrids maintain certain characteristics, such as the involvement of Zeb1 in EMT, despite processes that trigger tumor cell heterogeneity. Our data also indicate that the used breast cancer cell model may play an important role. The Zeb1-KO in MDA-MB-231 breast cancer cells was associated with re-induction of E-Cadherin, which is consistent with published data [41,42,43]. However, the Zeb1-KO in HS578T breast cancer cells was not associated with re-induction of E-Cadherin expression [29]. These findings point to breast cancer cell line-specific differences, which may need to be taken into account in studies on breast cancer and EMT.

## Data Availability

The data presented in this study are available upon reasonable request from the corresponding author.

## Supplementary Materials

The following supporting information can be downloaded at: https://www.mdpi.com/article/10.3390/ijms27188248/s1.

## Abbreviations

The following abbreviations are used in this manuscript:

ANOVA	analysis of variance
AR	androgen receptor
BSA	bovine serum albumin
CSCs	cancer stem cells
E	Epithelial
E/M	epithelial/mesenchymal
EMT	epithelial-to-mesenchymal transition
KO	knock-out
Krt5	cytokeratin 5
PBS	phosphate-buffered saline
PVDF	Polyvinyldifluoride
SDS-PAGE	sodium dodecylsulfate–polyacrylamide gel electrophoresis
S.E.M.	standard error of the mean
TBS-T	Tris-buffered saline with 1% (*v*/*v*) Tween 20
TNBC	Triple-negative breast cancer
