# Supplementary material for: Zeb1 Is a Determinant of EMT in Human MDA-MB-231 Breast Cancer Cells and M13-MDA231 Tumor Hybrids
Source: Int J Mol Sci. 2026 Sep 16;27(18):8248. doi: 10.3390/ijms27188248 (PMC13607604; doi:10.3390/ijms27188248)
Supplement: Supplementary file 1 [file ijms-27-08248-s001.zip › ijms-4455949-supplementary.pdf]

Table S1. Used antibodies.

Antibody	Clone; catalog number	Dilution
Anti-β-Actin (mouse monoclonal)	AC-15; #A5441	1:3000 in 5% BSA (w/v) in TBS-T
E-Cadherin (rabbit monoclonal)	24E10; #3195S	1:1000 in 5% BSA (w/v) in TBS-T
Cytokeratin-5 (rabbit monoclonal)	D4U8Q; #25807S	1:1000 in 5% BSA (w/v) in TBS-T
N-Cadherin (mouse monoclonal)	13A9; #14215S	1:1000 in 5% non-fat milk powder (w/v) in TBS-T
Snail (rabbit monoclonal)	C15D3; #3879S	1:1000 in 5% BSA (w/v) in TBS-T
Vimentin (rabbit polyclonal)	#3932S	1:1000 in 5% BSA (w/v) in TBS-T
Zeb1 (rabbit monoclonal)	D808D3; #3396S	1:1000 in 5% BSA (w/v) in TBS-T
Anti-mouse IgG, HRP-linked (horse polyclonal)	#7076S	1:1000 in 5% non-fat milk powder (w/v) in TBS-T
Anti-rabbit IgG, HRP-linked (horse polyclonal)	7074S	1:1000 in 5% non-fat milk powder (w/v) in TBS-T

The anti-β-Actin antibody was purchased from Merck KGaA, Darmstadt, Germany. All other antibodies were obtained from Cell Signaling Technology Europe B.V., Frankfurt am Main, Germany.
